# Development of *Candida* autochthonous starter for cigar fermentation *via* dissecting the microbiome

**DOI:** 10.3389/fmicb.2023.1138877

**Published:** 2023-02-24

**Authors:** Yun Jia, Yuanfa Liu, Wanrong Hu, Wen Cai, Zhaojun Zheng, Cheng Luo, Dongliang Li

**Affiliations:** ^1^Cigar Fermentation Technology Key Laboratory of China Tobacco, China Tobacco Industrial Co., Ltd., Chengdu, China; ^2^School of Food Science and Technology, Jiangnan University, Wuxi, China

**Keywords:** cigar fermentation, microbial community, functional microbes, *Candida* strains, bioaugmentation inoculation

## Abstract

**Introduction:**

The main goal of tobacco fermentation technology is to minimize the alkaloid content while improving flavor substance content.

**Methods:**

This study revealed the microbial community structure and their metabolic functions during cigar leaf fermentation by high-throughput sequencing and correlation analysis, and evaluated the fermentation performance of functional microbes based on in vitro isolation and bioaugmentation fermentation.

**Results:**

The relative abundance of *Staphylococcus* and *Aspergillus* increased first but then decreased during the fermentation, and would occupy the dominant position of bacterial and fungal communities, respectively, on the 21st day. Correlation analysis predicted that *Aspergillus*, *Staphylococcus* and *Filobasidium* could contribute to the formation of saccharide compounds, *Bacillus* might have degradation effects on nitrogenous substances. In particular, *Candida*, as a co-occurring taxa and biomarker in the later stage of fermentation, could not only degrade nitrogenous substrates and synthesize flavor substances, but also contribute to maintaining the stability of microbial community. Moreover, based on *in vitro* isolation and bioaugmentation inoculation, it was found that *Candida parapsilosis* and *Candida metapsilosis* could significantly reduce the alkaloids content and increase the content of flavor components in tobacco leaves.

**Discussion:**

This study found and validated the critical role of *Candida* in the fermentation of cigar tobacco leaves through high-throughput sequencing and bioaugmentation inoculation, which would help guide the development of microbial starters and directional regulation of cigar tobacco quality.

## 1. Introduction

Cigars are tobacco products rolled from tobacco leaves after air-curing, fermentation and aging, consisting of the wrapper, binder, and filler (Frankenburg, 1950). Fermentation is an important process for improving the quality of cigar leaves, the complex microbial communities on the surface and internal cigar leaves can secrete a variety of enzymes to degrade macromolecules (e.g., proteins, starch, cellulose) and harmful compounds (e.g., nicotine) in tobacco leaves, while generating various flavor components (e.g., terpenes, carbonyls), which ultimately lead to the formation of unique style characteristics of cigars (Zheng et al., 2022c). Cigar fermentation is mainly divided into natural and artificial fermentation, in which the moisture of tobacco leaves is first adjusted to around 30%, and then fermented under natural or artificially controlled conditions of temperature and humidity. In this process, tobacco leaves need to be regularly shuffled to improve homogeneity and avoid local overheating (Di Giacomo et al., 2007). Traditionally, cigar fermentation relies on the microorganisms existing in the raw materials and fermentation environment to perform enzymatic hydrolysis and microbial fermentation under natural temperature and humidity, i.e., natural fermentation (Zhang et al., 2013; Rivett and Bell, 2018; Li et al., 2020). However, the long production cycle (1–2 years), unstable quality, and external environmental influences restrict the development of the traditional fermentation industry of cigars. Compared with natural fermentation, artificial fermentation can be performed under controlled temperature (35–50°C) and relative humidity (70–85%), with relatively stable product quality and shorter fermentation cycles (4–8 weeks; Liu et al., 2021).

It is well known that tobacco alkaloids have potential carcinogenic effects and directly affect the bitterness and irritation of tobacco leaves, such as nicotine, anatabine and cotinine, of which nicotine accounts for more than 90% of the total (Kaminski et al., 2020; Rehder Silinski et al., 2020). Therefore, minimizing alkaloid content while improving flavor substance is the main goal of tobacco fermentation technology. Bioaugmentation with special strains (e.g., *Pseudomonas*, *Acinetobacter*, *Sphingomonas*) can not only degrade nicotine to improve safety, but also improve the flavor of tobacco products and shorten the fermentation cycle (Liu et al., 2015; Zheng et al., 2022a). Compared with other treatment methods, bioaugmentation has advantages in terms of cost, efficiency and sustainability. It will be helpful to develop functional autochthonous starters by analyzing the microbial communities in cigar fermentations and determining the effects of the microbial communities in a specific habitat on the quality of cigar leaves.

In recent years, high-throughput sequencing and multi-omics technologies have been widely applied in many microbial-related research fields, such as gut flora, fermentation foods and ecological environments (Cureau et al., 2021; Guo et al., 2022; Tigrero-Vaca et al., 2022). Previous studies have preliminarily analyzed the microbial community structure in tobacco fermentation products based on pure culture, denaturing gradient gel electrophoresis (PCR-DGGE) and high-throughput sequencing (Liu et al., 2021; Zheng et al., 2022b). As Li et al. (2020) followed the changes in the microbial communities of cigar leaves fermented at 45°C by amplicon sequencing, and found that the abundance of *Pseudomonas* increased with the extension of fermentation time, while that of *Sphingomonas* and *Methylobacterium* decreased. However, there is still a lack of systematic research on the fermentation mechanism of microbial communities and the development of functional starters in cigar tobacco fermentation. Recently, multi-omics (e.g., amplicon sequencing, metagenomics, metabolomics, culturomics) and statistical methods of correlation have provided opportunities to study the functions of microbial communities and a method to bridge the gap between phenotype and genotype in fermentation ecosystems (Vilanova and Porcar, 2016; Hall et al., 2018). Huang et al. determined functional microbes in vinegar fermentation by correlation analysis between microbial communities and flavor metabolites (Huang et al., 2022).

In this study, to dissect the microbial communities during cigar fermentation and their effects on quality, the successional patterns of microbial community structures were revealed by high-throughput sequencing. Then the metabolic functions of microbial communities were explored by correlation analysis, and the fermentation performance was evaluated based on *in vitro* isolation and bioaugmentation fermentation. The findings would facilitate the development of microbial starters and the improvement of cigar safety and quality.

## 2. Materials and methods

### 2.1. Chemicals and reagents

HPLC grade reagents used for chromatography were obtained from Thermo Fisher Scientific (Waltham, MA, United States). All standard compounds used in the study were from Sigma-Aldrich Co. Ltd. (St. Louis, MO, United States). The QuEChERS kit used for extraction with a 50 ml tube with 4 g MgSO_4_; 1 g NaCl; 1 g Na_3_Citrate; 0.5 g Na_2_HCitrate, purchased from Agilent (Santa Clara, CA, United States). Other commercial chemicals were of analytical grade and were purchased from Sinopharm Chemical Reagent Co., Ltd. (Shanghai, China).

### 2.2. Cigar tobacco leaves collection

In this study, the filler of Dexue 1 was used as the research object, and the tobacco leaves were all provided by China Tobacco Sichuan Industry Co., Ltd. (Sichuan, China). In the previous study, we optimized the fermentation process of Dexue 1 and obtained the optimal fermentation conditions (initial moisture of 30%, fermentation temperature of 30°C, relative humidity of 70%, and fermentation time of 35 days), under which the tobacco can reduce nicotine content and maintain better sensory quality. The specific fermentation was performed as follows, 5 kg tobacco leaves were moistened with sterile water until the moisture content reached 30%, then the tobacco leaves were evenly stacked in a constant temperature and humidity incubator at 30°C and 70% humidity, and three biological replicates were set. Samples were taken every 7 days during fermentation and stored at-80°C until detection.

### 2.3. Physicochemical composition analysis

The cigar leaves were ground to powder in liquid nitrogen with pestles. The contents of total sugar, reducing sugar, total alkaloid and total nitrogen were determined by API continuous flow instrument (Liu et al., 2021). The contents of flavor and alkaloid components were determined by gas chromatography–mass spectrometry (GC–MS). About 2 g of sample was accurately weighed into a 50 ml capped centrifuge tube, 10 ml of water was added, shaken until the sample was sufficiently infiltrated by water. After standing for 10 min, 10 ml acetonitrile and 50 μl phenylethyl acetate internal standard (9.06 mg/ml) were added, and shaken at 2000 rpm for 2 h. After the mixture was frozen at-20°C for 10 min, 4.0 g MgSO_4_; 1.0 g NaCl; 1.0 g Na_3_Citrate; 0.5 g Na_2_HCitrate were added, and immediately shaken to prevent clumping. Then, 1 ml supernatant was transferred into a 1.5 ml centrifuge tube, 0.15 g MgSO_4_ was added, shaken at 2000 rpm for 2 min, and centrifuged at 6000 rpm for 2 min to collect the supernatant in a sample bottle for further analysis.

The separation of compounds was carried out on DB-5MS column (60 m × 1.0 μm × 0.25 mm, Agilent Technology, Santa Clara, CA, United States). The initial temperature of the GC oven was 60°C, then ramped to 250°C at 2°C/min, and then held for 20 min after increasing to 290°C at 5°C/min. Helium was used as the carrier gas at the flow rate of 1.2 ml/min. The MS spectra were operated in the electron impact (EI) mode with an ion source temperature of 230°C and an ionization voltage of 70 eV. The mass scan range was 26–400 amu with a scanning rate of 0.2 scan/s. Compound identification was performed by matching the mass spectra with the NIST library (National Institute of Standards and Technology) and Wiley (NY, 320 k compounds, Ver. 6.0). Compounds quantification was calculated according to the ratio between the peak area of a particular compound and that of internal standard. According to the classification of precursors, metabolites are mainly classified into nicotine and its degradation products, degradation products of carotenoids, Maillard reaction products, degradation products of chlorophyll, and degradation products of cembranoids (Rodriguez-Bustamante et al., 2005).

### 2.4. Amplicon sequencing and analysis

The cigar leaves were ground to powder in liquid nitrogen with pestles. Next, DNA was extracted using the Powersoil DNA Isolation kit and DNeasy Power Max Soil Kit (MoBio, Carlsbad, CA, United States). The purity and integrity of extracted DNA were checked by Nanodrop 2000 (Thermo Fisher Scientific, Waltham, MA, United States) and agarose gel electrophoresis. The DNA was stored at-80°C before further analysis. The V3-V4 region of bacterial 16S rRNA genes and fungal ITS1 regions were amplified by primers 338-F/806-R and ITS1-F/ITS2-R with specific barcodes, respectively (Liu et al., 2018). Then, the library was sequenced on an Illumina HiSeq 2,500 platform by BGI Co., Ltd. (Wuhan, China). After sequencing, amplicon sequence variants (ASV_S_) were obtained by denoising using the Dada2 (Divisive Amplicon Denoising Algorithm) method in the software Qiime2. ASV sequences were annotated using the Silva bacterial database and the Unite fungal database. Alpha diversity index and beta diversity were calculated within Qiime2.

### 2.5. Isolation of *Candida* strains

To isolate *Candida* strains, 20 g of fermented tobacco leaves were mixed with 180 ml of sterile saline solution (0.85%) and homogenized with BagMixer for 2 min. Then the suspension was serially diluted 10-fold in sterile saline solution, and 100 μl dilution was spread on the surface of the yeast extract peptone dextrose (YPD) and potato dextrose agar (PDA) plates supplemented with ampicillin sodium, and incubated at 30°C for 3 days under aerobic and anaerobic conditions, respectively. Genomic DNA of single isolated strains was extracted according to the instruction of the TIANamp DNA kit (Tiangen, Beijing, China). The internal transcribed spacer (ITS) region was amplified with primers ITS1 and ITS4 as described previously (White et al., 1990). DNA sequencing of the PCR products was conducted by Sangon Biotech (Shanghai, China). After a BLAST search against the sequences^1^, the results were used to identify the isolates. By comparing the gene sequence similarity of the *Candida* isolate strains and the dominant ASV by NCBI, isolates with sequence similarity greater than 99% were taken as the representative strains of the dominant ASV.

### 2.6. Bioaugmentation of *Candida* strains

First, *Candida parapsilosis* strains and *Candida metapsilosis* strains were inoculated into PDB medium and grown at 30°C with shaking for 3 days, and then the cultures were centrifuged at 12000 rpm for 30 min to collect the cells. The cells were repeatedly suspended and centrifuged with sterile saline solution to ensure that there was no trace of medium in the inoculum. Next, different yeast strains were individually inoculated into 5 kg tobacco leaves with the initial cell density of each species adjusted to 1 × 10^6^ CFU/g. Then, tobacco leaves were evenly stacked in a constant temperature and humidity incubator at 30°C and 70% humidity for 35 days of fermentation. The non-inoculated sample was used as a control to evaluate the fermentation ability of different *Candida* strains by detecting the physicochemical indexes and flavor substances of the fermented tobacco leaves. All experiments were set up in three parallel.

### 2.7. Statistical analysis

Based on the differences in ASV_S_, partial least squares discrimination analysis (PLS-DA) was performed using mixOmics package in R (Version R-3.5.1; Takei et al., 2022). The linear discriminant analysis (LDA) effect size (LEfSe) algorithm was performed to identify the representative bacterial and fungal taxa at different fermentation periods. The spearman’s pairwise correlations were calculated simultaneously using corr.test function with psych package in R (Version R-3.5.1) to analyze the significance of the correlation. Significantly (*p*-value <0.05) high correlations (|*ρ*| > 0.6) were visualized *via* Cytoscape (version 3.6.1). Significance difference analysis and Z-score normalization were performed using SPSS (version 22.0, SPSS Inc., Chicago, IL, United States). Further statistical analysis and graphics were performed in EXCEL 2017 software (Microsoft Office, United States) and GraphPad Prism Software (version 8.0, GraphPad Software, San Diego, California, United States).

## 3. Results

### 3.1. Temporal changes of physicochemical metabolites

**Figure 1:**
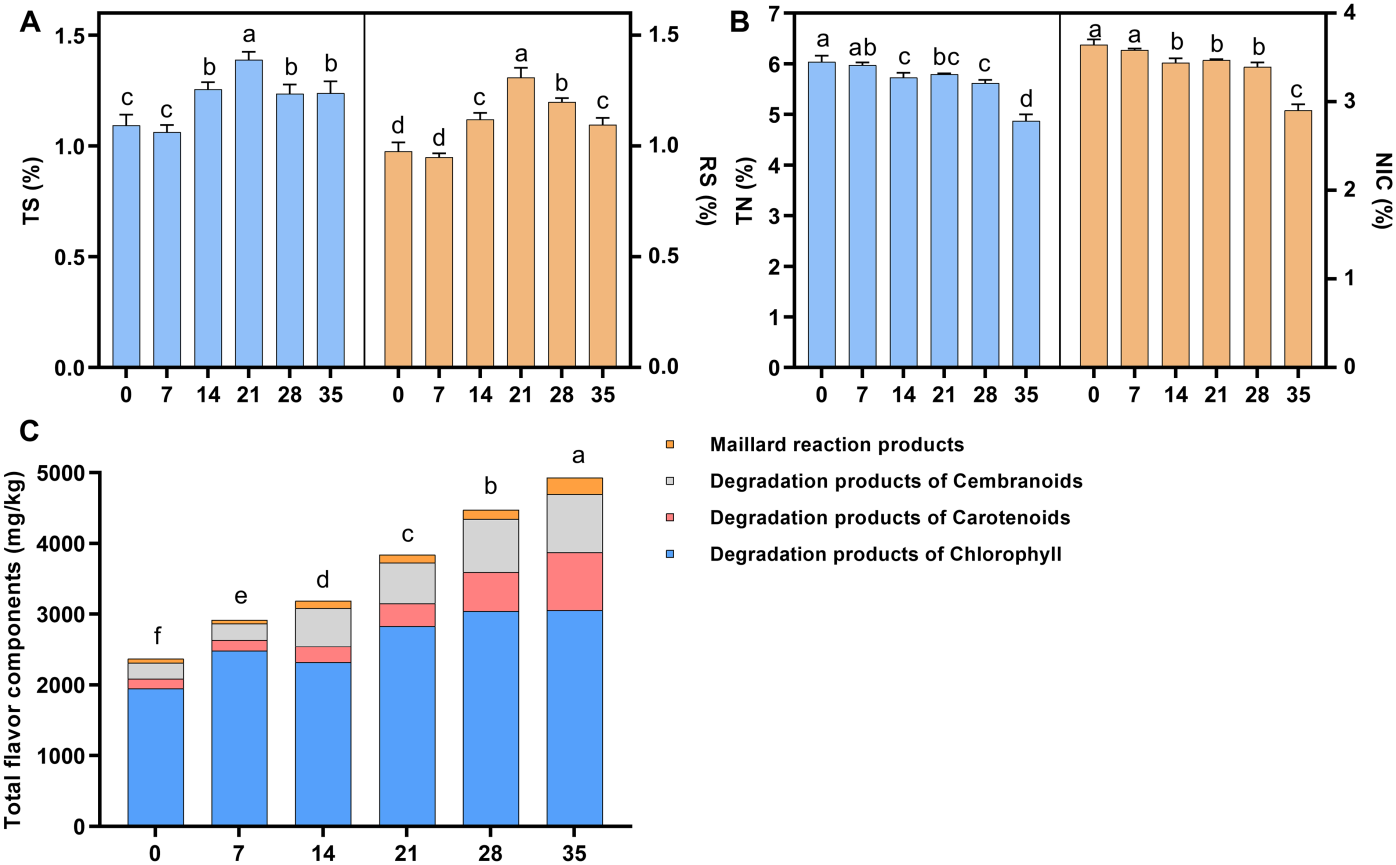
The changes of physicochemical metabolites during fermentation of cigar tobacco leaves. **(A)** Total sugar and reducing sugar content; **(B)** total nitrogen (TN) and alkaloid (NIC) content; **(C)** flavor component content. TS, total sugar; RS, reducing sugar; TN, total nitrogen; NIC, alkaloid. Letters indicated significant differences based on Turkey’s LSD, and the numbers 0, 7, 14, 21, 28, 35 indicated the fermentation days.

In this study, the contents of total sugar (TS), reducing sugar (RS), total nitrogen (TN), alkaloids (NIC) and total flavor components (FC) during cigar fermentation were analyzed (Figure 1). The contents of total sugar and reducing sugar are important indicators of the quality of tobacco leaves, they can form various flavor substances through a series of chemical reactions (Arihara et al., 2017; Rivera and Tyx, 2021). As shown in Figure 1A, with the development of fermentation, the starch in tobacco leaves was gradually degraded into small molecules of sugars by microorganisms, resulting in a gradual increase in the content of total sugar and reducing sugar. However, the content of total sugar and reducing sugar decreased in the later stage of fermentation due to microbial utilization and the Maillard reaction (Liu et al., 2021). The content of total nitrogen and alkaloids decreased by 12.6 and 26.1%, respectively, during the fermentation (Figure 1B), which might be due to the gradual degradation of these substances into small molecules of amino acids, organic acids and volatile ammonia during fermentation by microorganisms (Frankenburg, 1950; Di Giacomo et al., 2007; Vigliotta et al., 2007; Liu et al., 2015). As shown in Figure 1C, the total content of flavor components gradually accumulated during fermentation, and the detected flavor components mainly included degradation products of carotenoids, Maillard reaction products, degradation products of chlorophyll and degradation products of cembranoids, which could form a unique flavor of cigars (Banozic et al., 2021).

### 3.2. Structure and succession of microbial community

Bacterial and fungal communities during fermentation were analyzed by amplicon sequencing. As shown in Supplementary Figures S1A,C, the microbial richness of bacteria and fungi decreased first and then increased with fermentation. This might be due to the gradual extinction of some microbes affected by the environment (e.g., high nicotine content) in the early stage of fermentation, leading to a decrease in microbial richness, after which microbial richness gradually increased as the enrichment of tolerant microbes (Chao and Bunge, 2002; Liu et al., 2021). PLSDA analysis showed that both bacterial and fungal communities changed significantly during the fermentation, in which bacterial community changed significantly during the 21–35 days of fermentation, while the fungal community changed significantly during the 0–7 days and 28–35 days of fermentation (Supplementary Figures S1B,D).

**Figure 2:**
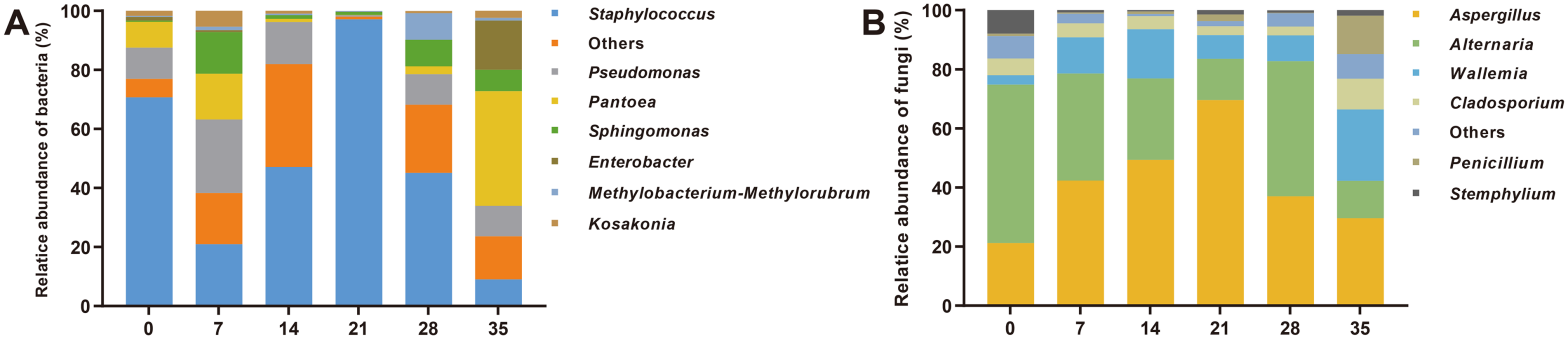
Changes in microbial community structure during fermentation of cigar tobacco leaves. **(A)** Bacteria; **(B)** fungi. The numbers 0, 7, 14, 21, 28, 35 indicated the fermentation days.

Dominant microbiota with high abundance (average relative abundance >1%) is generally considered as an important component of traditional fermentation, which might play key roles in biological processes (Wolfe et al., 2014; Huang et al., 2018). According to the statistical analysis of the samples (Figure 2), it was found that the dominant bacterial genera mainly include *Staphylococcus* (47.8%), *Pseudomonas* (13.0%), *Pantoea* (11.7%), *Sphingomonas* (5.0%), *Enterobacter* (2.7%), *Kosakonia* (2.2%) and *Methylbacterium methylarum* (1.6%). Among them, the relative abundance of *Staphylococcus* increased first and then decreased during the fermentation (except for the high abundance on day 0, due to the introduction of the external environment), reaching a maximum of 96.3% on the 21st day, and occupying the dominant position of the bacterial community. The change trends of *Pseudomonas*, *Pantoea* and *Enterobacter* were opposite to that of *Staphylococcus*, decreasing first and then increasing. The dominant fungal genera mainly included *Aspergillus* (41.6%), *Alternaria* (31.6%), *Wallemia* (12.1%), *Cladosporium* (5.3%), *Penicillium* (2.9%) and *Stemphyllium* (2.2%). Among them, the relative abundance of *Aspergillus* increased first and then decreased, reaching the highest 69.8% on the 21st day, which was the dominant fungal genus. LEfSe analysis was used to further determine the biomarker before and after fermentation, and Proteobateria (*Pantooea*, *Enterobacter*, *Stenotrophomonas*), Basidiomycota (*Wallemia*) and Ascomycota (*Penicillium*, *Candida*) were found to be the main differential microbes after fermentation (Supplementary Figure S2; Supplementary Table S1).

### 3.3. Microbial function prediction

**Figure 3:**
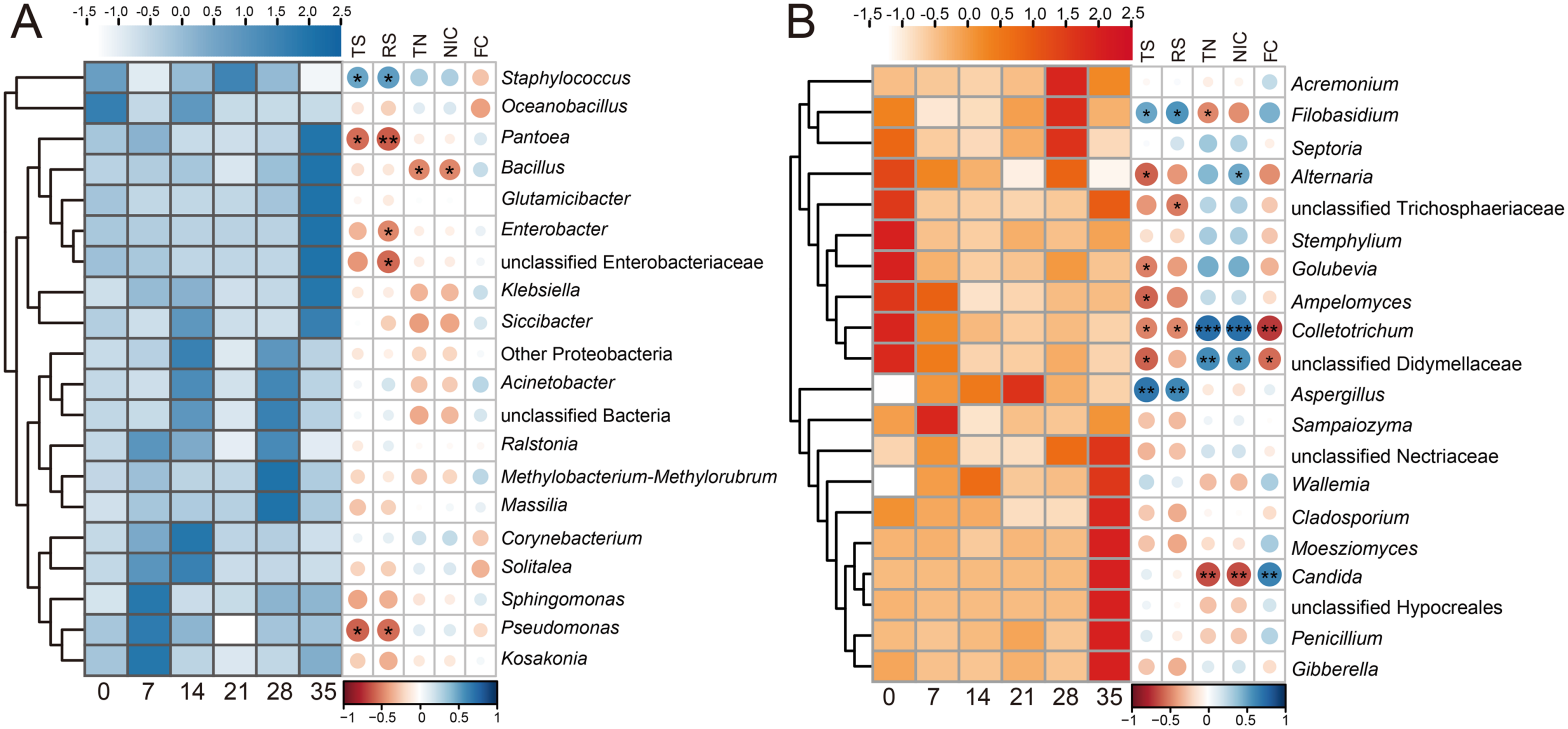
Heatmaps showing the succession of Top20 genera and the correlations between each genus and fermentation indicators during cigar fermentation. **(A)** Bacteria; **(B)** fungi. The relative abundance of each microbial genus was normalized using Z-score. Succession in microbial genera is shown on the left, color is positively correlated with relative abundance. The right side shows the Spearman correlation between each genus and fermentation indicators, the correlation coefficient is represented by the color and size of the circles, dark blue indicates positive correlation, and dark red indicates negative correlation. *p < 0.05; **p < 0.01; ***p < 0.001. TS, total sugar; RS, reducing sugar; TN, total nitrogen; NIC, alkaloid. The numbers 0, 7, 14, 21, 28, 35 indicated the fermentation days.

Microbial community succession is often accompanied by the transformation of metabolic substances, and the functional potential of microbial communities could be preliminarily predicted through correlation analysis. Figure 3 showed that *Aspergillus*, *Staphylococcus*, and *Filobasidium* were positively correlated with total sugar and reducing sugar contents, which might contribute directly or indirectly to the production of saccharide compounds. *Bacillus* and *Candida* were negatively correlated with total nitrogen and alkaloid contents, which might have degradation effects on nitrogenous substances (e.g., proteins, alkaloids). Besides, *Candida* was positively correlated with the content of total flavor components, which might have the ability to synthesize flavor substances.

In addition, recent studies suggested that co-occurring taxa could have a considerable impact on microbial community structure and function, regardless of their abundance (Banerjee et al., 2018). Network analysis facilitates the exploration of many interconnected correlations simultaneously and has become an effective tool for identifying co-occurring taxa in complex ecosystems (Wang et al., 2019). Based on the microbial network analysis, complex correlations between different genera were found (Supplementary Figure S3). A total of 93 nodes (48 bacterial nodes and 45 fungal nodes) and 185 pairs of significant and robust edges (167 pairs of positive correlation and 18 pairs of negative correlation) were obtained (|*ρ*| > 0.6, *p* < 0.05). In this study, the nodes with degree above 10 were defined as co-occurring taxa (Wang et al., 2019). As shown in Supplementary Table S2, four fungal genera (*Cryptococcus*, unclassified Catabotrydaceae, *Candida*, unclassified Ustilaginaceae) were identified as co-occurring taxa. For example, *Candida* might play a key role in complex fermentation systems, although it only accounted for 0.42% of the total fungal abundance.

### 3.4. Isolation and bioaugmentation fermentation of *Candida*

Statistical methods indicated that *Candida*, as a co-occurring genus and biomarker in the late fermentation period, could not only degrade nitrogenous substances and synthesize flavor substances, but also play a key role in maintaining the stability of microbial community structure and function. Therefore, *Candida* strains from cigar tobacco leaves were isolated, and representative species with high relative abundances were obtained (Table 1). Species identification results indicated that *C. parapsilosis* and *C. metapsilosis* were the major *Candida* species, and their relative abundances accounted for 74.0 and 13.9% of *Candida* spp., respectively.

**Table 1:** Isolation of *Candida* strains*^a^*.

Dominant ASVs	Relative abundance of dominant ASV in *Candida* (%)	Isolates	Similarity (%)
ASV27	74.0%	*Candida parapsilosis*	100
ASV65	13.9%	*Candida metapsilosis*	100

^a^Sequence similarity between the isolate and dominant ASV was compared by NCBI.

**Figure 4:**
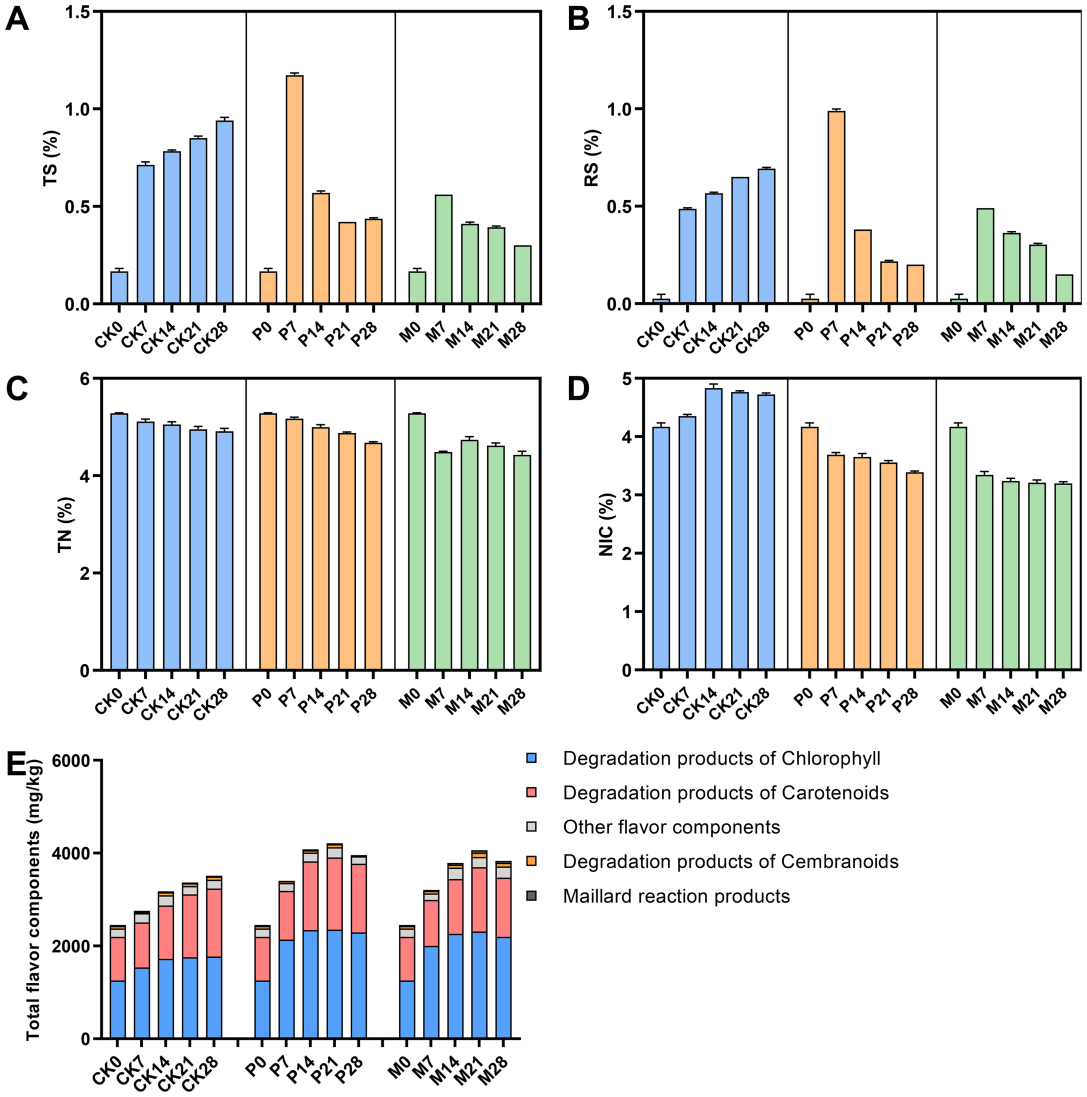
Changes of physicochemical metabolite during bioaugmentation fermentation by different *Candida* strains. **(A)** Total sugars content; **(B)** Reducing sugar content; **(C)** Total nitrogen content; **(D)** Total alkaloids content; **(E)** Total flavor components content. TS, total sugar; RS, reducing sugar; TN, total nitrogen; NIC, alkaloid. CK0-CK28: Control group; P0-P28: *C. parapsilosis* group; M0-M28: *C. metapsilosis* group, and the numbers 0, 7, 14, 21, 28 indicated the fermentation days.

**Figure 5:**
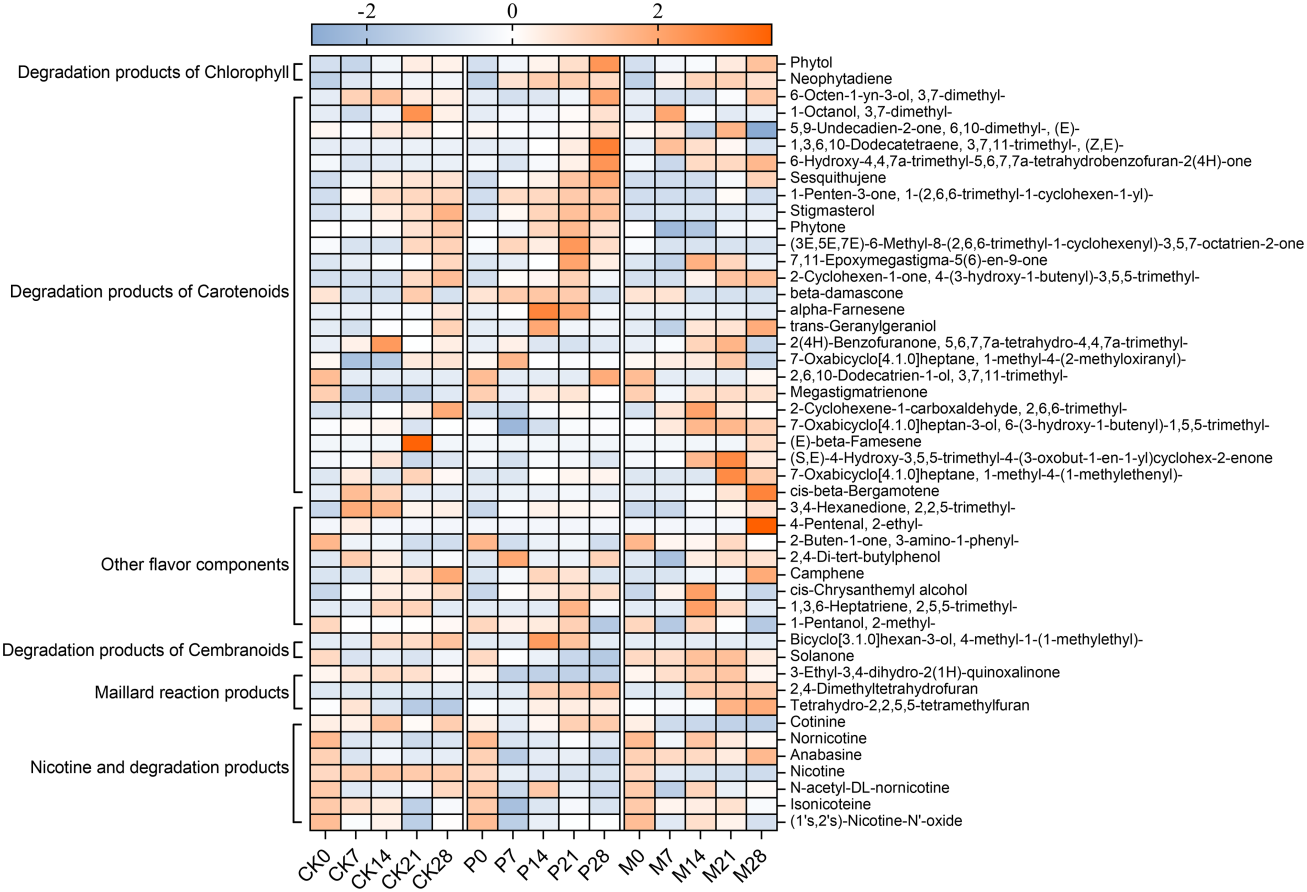
Change in the contents of flavor compounds and alkaloids during bioaugmentation fermentation of *Candida* strains. The peak area of each compound was normalized using Z-score. The color intensity was proportional to the concentration of compounds. CK0-CK28: Control group; P0-P28: *C. parapsilosis* group; M0-M28: *C. metapsilosis* group, and the numbers 0, 7, 14, 21, 28 indicated the fermentation days.

Next, the isolated *C. parapsilosis* and *C. metapsilosis* were separately inoculated into cigar tobacco leaves, and the physicochemical metabolites after bioaugmentation fermentation were detected and analyzed. As shown in Figures 4A,B, the contents of total sugar and reducing sugar gradually increased in the fermentation of control (CK) group, while the content in *C. parapsilosis* (P) group and *C. metapsilosis* (M) group reached the highest on the 7th day, but gradually decreased with the fermentation. Among them, the contents of total sugars and reducing sugars were higher in group P than in group M. The total nitrogen content showed a decreasing trend during fermentation in the CK, P and M groups (Figure 4C). The total alkaloid content in groups P and M showed a downward trend and was significantly lower than that in group CK (Figure 4D). Qualitative and quantitative analysis of alkaloids in different fermentation groups by GC–MS detected seven alkaloids, including cotinine, nornicotine, nicotine, N-acetyl-DL-nornicotine, isonicoteine, (1’s, 2’s)-nicotine-N′-oxide (Figure 5). Among them, nicotine accounted for 92.7–98.0% of the total alkaloid content, showing a first rising and then decreasing trend in the CK group, and a downward trend in groups P and M.

In addition, the content of total flavor components in the CK group gradually accumulated during fermentation, while in groups P and M, it reached the highest level on the 21st day of fermentation and then gradually declined (Figure 4E). The total flavor components contents of these three groups were ranked from high to low as: P > M > CK. Figure 5 showed the variation trends of 40 flavor components contents during fermentation. The results showed that the contents of chlorophyll degradation products and carotenoids degradation products in group P were higher than those in group CK, and reached the highest levels on the 21st to 28th days of fermentation. Among them, the degradation products of chlorophyll and carotenoids in group P accounted for 37.6–49.6% and 24.3–28.7% of the total content of flavor components, respectively. In addition, the products of the degradation of cembranoids and Maillard reactions were higher in the M group than in the CK group.

## 4. Discussion and conclusions

The dominant microbiota is generally considered to play an important role in biological processes (Huang et al., 2018). Amplicon sequencing revealed seven bacterial (*Staphylococcus*, *Pseudomonas*, *Pantoea*, *Sphingomonas*, *Enterobacter*, *Kosakonia* and *Methylobacterium methylorubrum*) and six fungal genera (*Aspergillus*, *Alternaria*, *Wallemia*, *Cladosporium*, *Penicillium* and *Stemphyllium*) were the dominant genera during cigar fermentation (Figure 2). Among them, the relative abundances of *Staphylococcus* and *Aspergillus* increased first and then decreased during fermentation, and occupied the dominant position of bacterial and fungal communities on the 21st day, respectively. Although the microbial communities of cigar samples varied by region, variety, and fermentation process, the dominant genera were universally similar, mainly including *Staphylococcus*, *Aspergillus*, *Pseudomonas*, *Pantoea*, *Wallemia* (Zheng et al., 2022a).

Microbial function prediction indicated that *Aspergillus*, *Staphylococcus*, *Filobasidium*, *Bacillus*, and *Candida* might play different key roles in cigar tobacco leaves fermentation (Figure 3). *Aspergillus*, *Staphylococcus* and *Filobasidium* have been reported to produce enzymes necessary for starch degradation, i.e., with debranching activity α-Amylase and glucoamylase, thereby leading to increases of total and reducing sugar contents and providing precursor materials for subsequent growth of other microorganisms (Mot and Verachtert, 1985; Lakshmi et al., 2013; Jia et al., 2020). *Bacillus* and *Candida* not only have high environmental tolerances, but also have high utilization capacities of nitrogen-containing substances, which could reduce the irritation of tobacco leaves (Viana et al., 2010; Liu et al., 2015; Ruan et al., 2019). *Candida*, as a biomarker in the late stage of fermentation, might contribute to the synthesis of flavor substances and lead to the formation and accumulation of flavor substances. The ability of *Candida* to synthesize flavor components has been demonstrated in many studies and successfully applied to traditional fermented foods such as liquor, coffee, soy sauce, and broad bean paste (Wang et al., 2019; Bressani et al., 2021; Jiang et al., 2021; Lu et al., 2021; Tigrero-Vaca et al., 2022).

In addition, microbial network analysis indicated that rare fungal genera (*Cryptococcus*, unclassified Catabotrydaceae, *Candida* and unclassified Ustilaginaceae), as co-occurring taxa, might be important in maintaining the structural and functional stability of microbial community (Li et al., 2018). Although their relative abundance only accounted for 0.01–0.42% of the fungal community, they might affect the ability of the community to degrade specific substrates (Rivett and Bell, 2018). And positive correlations accounted for 90.3% of the total interaction relationships, these positive interactions implied the growth promotion among microorganisms, and the complementation of metabolic pathways might be responsible for the coexistence of related microorganisms (Sieber et al., 2012; Widder et al., 2016). In general, the correlations between microorganisms and physicochemical metabolites, as well as the complex interactions between microorganisms, collectively determined the ultimate flavor of cigars (Singh et al., 2017).

It is well known that high levels of alkaloids not only directly affect the bitterness, astringency, and irritation of tobacco leaves, but also affects human health (Kaminski et al., 2020). So, the main goal of tobacco fermentation technology is to minimize alkaloid content while improving flavor substances. Previous analysis showed that *Candida* not only had a degradation effect on nitrogenous substances, but also contributed to the synthesis of flavor substances. Therefore, *Candida* strains were isolated for their fermentation performance evaluation in cigar tobacco leaves. Bioaugmentation fermentation showed that predominant *Candida* strains (*C. parapsilosis* and *C. metapsilosis*) not only decreased total nitrogen and alkaloids in cigar tobacco leaves but also significantly increased the content of flavor components. Among them, *C. parapsilosis* could elevate the content of chlorophyll degradation products and carotenoid degradation products. As one of the most abundant components in tobacco, neophytadiene, a chlorophyll degradation product, has the effects of increasing flavor and reducing irritation (Li et al., 2020). β-Damascone could increase fruity, floral as well as flavor complexity, and megastigmatrienone has a nutty and woody flavor (H. Zhang et al., 2021; Zhu et al., 2021). While *C. metapsilosis* could increase the content of cembranoids degradation and Maillard reaction products. Maillard reaction products, such as 2,4-dimethyltetrahydrofuran and tetrahydro-2,2,5,5-tetramethylfuran, which have the flavor of nuts, smoke, and baking, contribute significantly to the flavor of tobacco leaves (Koné et al., 2016). Cembranoids degradation products, such as solanone, associated with fruity flavor, can enhance the mellow and aftertaste of cigar tobacco leaves (Zhang et al., 2013; Zheng et al., 2022a).

In conclusion, this study revealed the microbial structure and succession pattern during the fermentation of cigar tobacco leaves by high-throughput sequencing. The relative abundances of *Staphylococcus* and *Aspergillus* both increased first and then decreased during fermentation, and occupied the dominant position of bacterial and fungal communities on the 21st day, respectively. Then through correlation analysis, we found that *Aspergillus*, *Staphylococcus*, and *Filobasidium* might contribute to the production of saccharide compounds, *Bacillus* might have a degradation effect on nitrogenous substances. In particular, *Candida* as a co-occurring genus, could not only degrade nitrogenous substances and synthesize flavor substances, but also play a key role in maintaining the stability of microbial community structure and function. Therefore, the fermentation performance of *Candida* strains (*C. parapsilosis* and C. *metapsilosis*) was evaluated based on *in vitro* isolation and bioaugmentation fermentation. The results showed that bioaugmentation of *C. parapsilosis* and *C metapsilosis* could not only reduce the total nitrogen and alkaloids in cigar tobacco leaves to improve safety, but also improve the flavor and shorten the fermentation cycle. Next, we will further reveal the effect of *C. parapsilosis* and *C metapsilosis* as starters on microbial community and sensory quality of cigar tobacco leaves, to guide the development of autochthonous starters and scale-up production.

## Data availability statement

The datasets presented in this study can be found in online repositories. The names of the repository/repositories and accession number (s) can be found in the article/Supplementary material.

## Author contributions

YJ: conceptualization, methodology, and writing—original draft. YL: supervision and writing—review and editing. WH: methodology. ZZ and CL: investigation. WC: validation. DL: project administration and writing—review and editing. All authors contributed to the article and approved the submitted version.

## Funding

The authors declare that this study received funding from China National Tobacco Corporation. The funder was not involved in the study design, collection, analysis, interpretation of data, the writing of this article, or the decision to submit it for publication.

## Conflict of interest

Authors YJ, WH, WC, CL and DL were employed by China Tobacco Industrial Co., Ltd.

The remaining authors declare that the research was conducted in the absence of any commercial or financial relationships that could be construed as a potential conflict of interest.

## Publisher’s note

All claims expressed in this article are solely those of the authors and do not necessarily represent those of their affiliated organizations, or those of the publisher, the editors and the reviewers. Any product that may be evaluated in this article, or claim that may be made by its manufacturer, is not guaranteed or endorsed by the publisher.

## Footnotes

^1^ http://www.ncbi.nlm.nih.gov/BLAST/
