# Supplementary material for: Development of Candida autochthonous starter for cigar fermentation via dissecting the microbiome
Source: Front Microbiol. 2023 Feb 24;14:1138877. doi: 10.3389/fmicb.2023.1138877 (PMC9998997; doi:10.3389/fmicb.2023.1138877)
Supplement: Supplementary file 1 [file Data_Sheet_1.pdf]

frontiers

Supplementary Material

Development of *Candida* autochthonous starter for cigar fermentation via dissecting the microbiome

Yun Jia[1,2], Yuanfa Liu[2], Wanrong Hu[1], Wen Cai[1], Zhaojun Zheng[2], Cheng Luo[1] and Dongliang Li[1,*]

*** Correspondence:** Dongliang Li: 360188228@qq.com

1 Supplementary Figures and Tables

1.1 Supplementary Figures

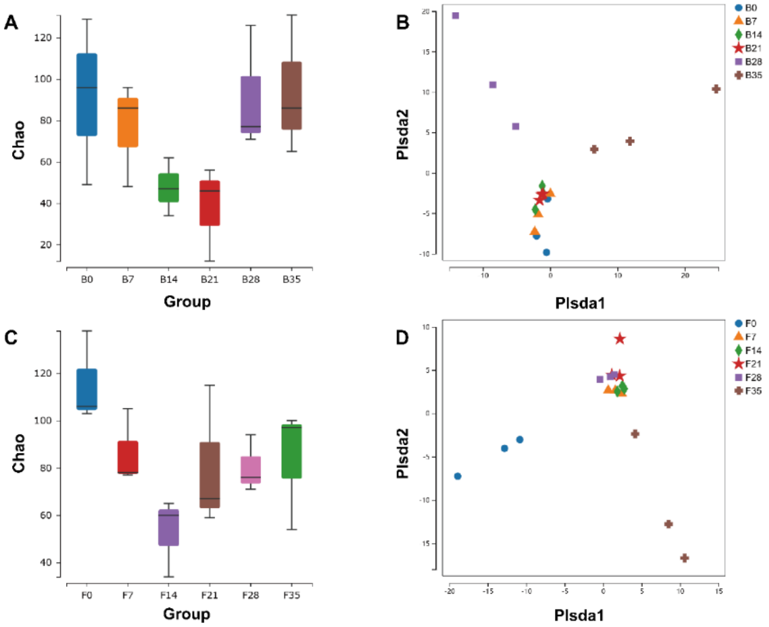

Supplementary Figure 1. Microbial diversity analysis. (A) bacterial chao index (B) bacterial PLSDA analysis (C) fungal chao index (D) fungal PLSDA analysis

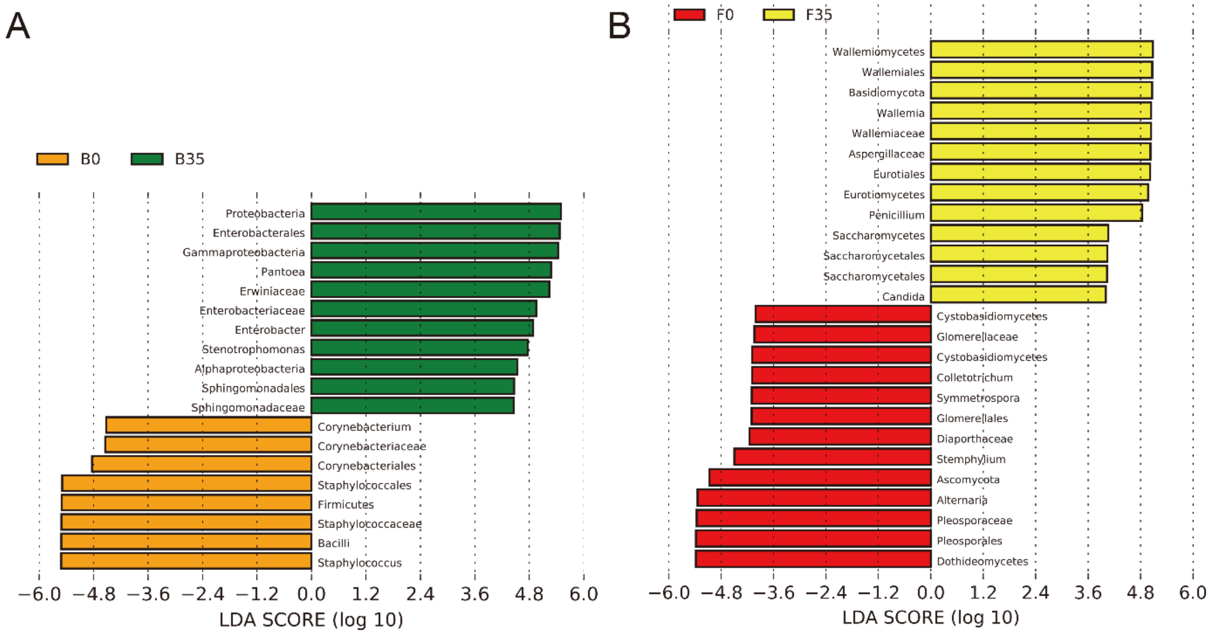

Supplementary Figure 2. Microbial biomarkers before and after fermentation. (A) bacteria (B) fungi

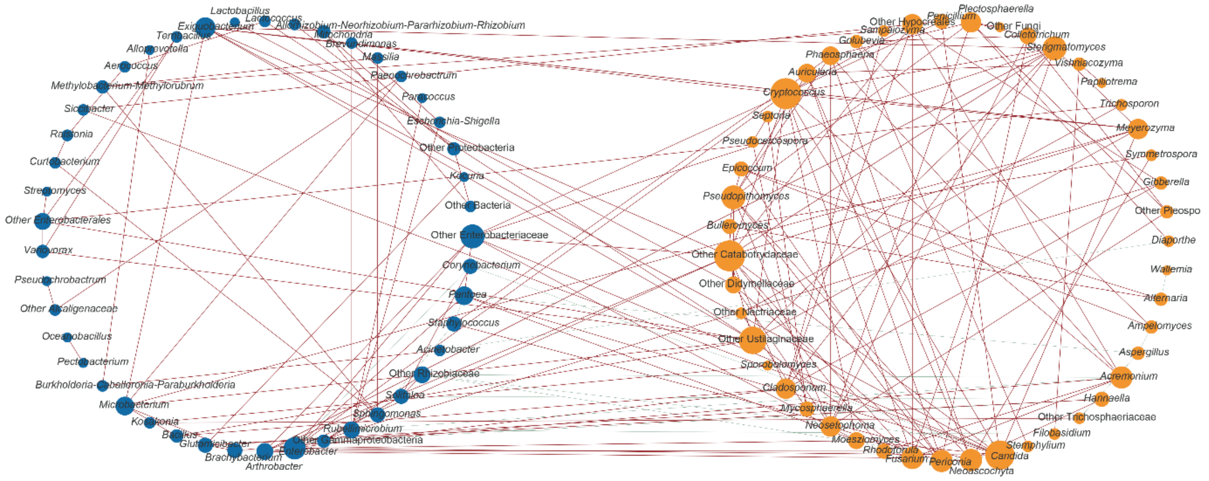

Supplementary Figure 3**.** Correlation network of co-occurrence and co-exclusion relationships between different genera. Statistically significant (P < 0.05) Spearman's correlation coefficient ($|\rho|$ > 0.6) indicates the robust correlations. The size of nodes indicates the degree of connections. Blue and orange nodes indicate bacteria and fungi respectively. Edge thickness represents the proportional to the value of Spearman's correlation. Green and red edges indicate negative and positive interaction between nodes**.**

1.2 Supplementary Tables

Supplementary Table 1. Microbial biomarkers before and after fermentation

Sample	Kingdom	Phylum	Class	Order	Family	Genus
B0	Bacteria	Actinobacteriota	Actinobacteria	Corynebacteriales	Corynebacteriaceae	*Corynebacterium*
B0	Bacteria	Firmicutes	Bacilli	Staphylococcales	Staphylococcaceae	*Staphylococcus*
F0	Fungi	Ascomycota	Sordariomycetes	Glomerellales	Glomerellaceae	*Colletotrichum*
F0	Fungi	Basidiomycota	Cystobasidiomycetes	Cystobasidiomycetes	Symmetrosporaceae	*Symmetrospora*
F0	Fungi	Ascomycota	Dothideomycetes	Pleosporales	Pleosporaceae	*Stemphylium*
F0	Fungi	Ascomycota	Dothideomycetes	Pleosporales	Pleosporaceae	*Alternaria*
B35	Bacteria	Proteobacteria	Gammaproteobacteria	Enterobacterales	Erwiniaceae	*Pantoea*
B35	Bacteria	Proteobacteria	Gammaproteobacteria	Enterobacterales	Enterobacteriaceae	*Enterobacter*
B35	Bacteria	Proteobacteria	Gammaproteobacteria	Xanthomonadales	Xanthomonadaceae	*Stenotrophomonas*
F35	Fungi	Basidiomycota	Wallemiomycetes	Wallemiales	Wallemiaceae	*Wallemia*
F35	Fungi	Ascomycota	Eurotiomycetes	Eurotiales	Aspergillaceae	*Penicillium*
F35	Fungi	Ascomycota	Saccharomycetes	Saccharomycetales	Saccharomycetales	*Candida*

Supplementary Table 2. Co-occurring genera[a, b]

Genera	Degree	Relative abundance
Cryptococcus	13	0.07%
unclassified Catabotrydaceae	13	0.11%
Candida	12	0.42%
Unclassified Ustilaginaceae	11	0.01%

[a] The number represented the number of genera coexisting with other genera.

[b] Genera with degree above 10 were defined as co-occurring genera.

2 Supplementary data

The raw sequence data reported in this paper have been deposited in the Genome Sequence Archive in the Beijing Institute of Genomics (BIG) Data Center, Chinese Academy of Sciences, under accession numbers CRA014345 and CRA014351 that are publicly accessible at https://bigd.big.ac.cn/gsa.
